# Can patterns of chromosome inversions in *Drosophila pseudoobscura* predict polyandry across a geographical cline?

**DOI:** 10.1002/ece3.1165

**Published:** 2014-07-10

**Authors:** Paul Herrera, Michelle L Taylor, Alison Skeats, Tom A R Price, Nina Wedell

**Affiliations:** 1 Biosciences, University of ExeterCornwall Campus, Penryn, TR10 9FE, U.K; 2 Institute of Integrative Biology, University of LiverpoolLiverpool, L69 7ZB, U.K

**Keywords:** Chromosome inversion, *Drosophila pseudoobscura*, karyotype, polyandry

## Abstract

Female multiple mating, known as polyandry, is ubiquitous and occurs in a wide variety of taxa. Polyandry varies greatly from species in which females mate with one or two males in their lifetime to species in which females may mate with several different males on the same day. As multiple mating by females is associated with costs, numerous hypotheses attempt to explain this phenomenon. One hypothesis not extensively explored is the possibility that polyandrous behavior is captured and “fixed” in populations via genetic processes that preserve the behavior independently of any adaptive benefit of polyandry. Here, we use female isolines derived from populations of *Drosophila pseudoobscura* from three locations in North America to examine whether different female remating levels are associated with patterns of chromosome inversions, which may explain patterns of polyandry across the geographic range. Populations differed with respect to the frequency of polyandry and the presence of inversion polymorphisms on the third chromosome. The population with the lowest level of female remating was the only one that was entirely comprised of homokaryotypic lines, but the small number of populations prevented us investigating this relationship further at a population level. However, we found no strong relationship between female remating levels and specific karyotypes of the various isolines.

## Introduction

Multiple mating by females, known as polyandry, is a pervasive feature of many species, which challenges the traditional view that females are the choosy, monogamous sex (Williams 1966; Trivers 1972). The level of polyandry varies greatly, ranging from species in which females mate solely with one male to species in which a female may mate with hundreds of different males throughout her lifetime (Reguera et al. 2004). Such a ubiquitously occurring behavior as polyandry demands an explanation as it has broad implications, being the driving mechanisms of several aspects of sexual selection such as sperm competition (Simmons 2005) and postcopulatory female choice (Eberhard 1996), as well as affecting speciation, sexual conflict, and gene flow between populations (Burke 1989; Zeh et al. 1997; Arnqvist and Rowe 2005).

Numerous hypotheses have been proposed to explain the adaptive significance of polyandry that fall into two nonmutually exclusive categories: direct and genetic benefits (Reynolds 1996; Arnqvist and Nilsson 2000; Jennions and Petrie 2000). By far, genetic benefits have been more challenging to demonstrate experimentally (Baker et al. 2001). Females may benefit from mating multiply by decreasing genetic incompatibility between mates (Tregenza and Wedell 1998; Newcomer et al. 1999), creating sperm competition which promotes fertilization by genetically superior males (Olsson et al. 1996; Hosken et al. 2003) as well as by reducing vulnerability to harmful selfish genetic elements (Price et al. 2008; Wedell 2013). Alternatively, polyandry may present no adaptive value for females and arises either due to a genetic correlation between the sexes in mating rate (Halliday and Arnold 1987; Arnold and Halliday 1988) or from incomplete female control over mating rate (Ridley 1990; Rowe et al. 1994). However, polyandry is also associated with costs, such as increased transmission of sexual infections (Thrall et al. 2000), risk of physical damage (Kamimura 2007) or death (Parker 1970), and if nothing else a waste of time and energy (Arnqvist 1997). Hence, there are generally expected to be advantages to polyandry that counteract such costs.

One genetically based hypothesis not hitherto explored is whether polyandry is captured and fixed in populations by artifacts of the genetic architecture such as chromosome inversions. Inversions are sections of chromosome that have become inverted relative to the homologous chromosome so that they no longer match. The evolutionary consequence of this is that sections of chromosomes do not recombine during meiosis and become “preserved” across generations, carrying with them any traits locked within these genomic sections. If polyandrous behavior were to become captured within a section of inverted chromosome in this way, patterns of polyandry could be explained by the benefits of other genes captured within such inversions, rather than any direct adaptive benefits of polyandry itself. Alternatively, alleles controlling female remating behavior might be associated with inversions because inversions allow the capture of alleles for traits that promote fitness under a particular remating regime. Hence, an inversion associated with high polyandry might carry coadapted alleles that reduce harm caused by multiple mating or reduce the impact of sexually transmitted infections. Some important behavioral traits are known to be linked to inversions in natural insect populations, such as the gene *Gp-9* that is associated with an inversion in the fire ant, S*olenopsis invicta*. This gene has a major effect on the likelihood that queens are accepted in multiple-queen colonies (Keller and Ross 1999). Furthermore, Lawson et al. (2012) recently showed that facultative polyandry in queens has a genetic basis, and it is dependent on the male *Gp-9* genotype. Thus, although the genetic basis of polyandry is poorly understood, there is evidence that inversions may be linked to specific gene complexes involved in regulating female multiple mating.

To test the hypothesis that the genetic control of female remating behavior may be associated with inversions, we need a species that has highly heritable variation in polyandry and also shows substantial variation in inversions. The fly *Drosophila pseudoobscura* fulfills both these criteria. *D. pseudoobscura* is a small dipteran which is naturally found in woodland in North and Central America (Jaenike 2001). Previous work has found significant genetic variation in remating propensity in females from different populations (Price et al. 2014) and from different families (Price et al. 2011). There is also evidence for a latitudinal cline in polyandry across North America that may help to regulate the spread of a sex ratio distorting selfish genetic element (*SR*) (Price et al. 2014). *D. pseudoobscura* have four pairs of chromosomes in addition to the sex chromosomes (Tan 1935). The third chromosome harbors a rich polymorphism for overlapping paracentric inversions (Dobzhansky and Sturtevant 1938) with over thirty gene arrangements already identified (Schaeffer et al. 2003), ten of which are abundant and widely dispersed within North America (Powell 1992). The principal role of the inversions in the evolution of these flies, when in heterozygous condition, is perceived to be the suppression of crossing over between gene complexes that have reached an adaptive equilibrium (Kastritsis and Crumpacker 1966). Owing to this suppression, the genetic identity of linked gene complexes may be maintained for many generations as a “supergene” (Hartl 1998). Frequencies of the common gene arrangements in some populations of *D. pseudoobscura* undergo predictable seasonal changes, which provide clear evidence that these inversions are under strong selection (Dobzhansky 1970; Lewontin 1974; Powell 1992). Apart from seasonal changes, the frequencies of chromosomal polymorphisms in *D. pseudoobscura* also exhibit latitudinal and altitudinal clines, as well as long-term variations as a result of environmental changes (Dobzhansky 1944; Anderson 1989).

Alleles or genes controlling polyandry might be associated with one or more variants of this third chromosome inversion in *D. pseudoobscura*. However, inversion heterozygotes are very common in natural *D. pseudoobscura* populations and have often been demonstrated to show a fitness advantage relative to homokaryotypes (e.g., Dobzhansky 1951; Spiess 1962; but see Nickerson and Druger 1973). Several hypotheses suggest that polyandry in general should be commoner in females where the costs of remating are lower. Higher fitness females may have lower costs to polyandry, and so it is possible that polyandry is not associated with any one inversion, but instead is the behavior displayed by high fitness heterokaryotypic females, providing another potential explanation for the variation in polyandry across the species range.

In this study, we firstly confirm variation among female isolines in the propensity to remate in three North American populations of *D. pseudoobscura* previously shown to differ in remating rates and investigate whether these differences are associated with specific inversion karyotypes. We also examine whether polyandry is associated with heterokaryotype chromosome inversions, rather than homokaryotypes.

## Materials and Methods

### Fly stocks

We established three separate laboratory populations of *D. pseudoobscura* from approximately 100 females collected from each of three natural US populations in July 2012 at Lewistown, Montana (47°04′47″ N; 109°16′53″ W), collected between 1287 m and 1433 m above sea level, Show Low, Arizona (34°07′37″ N; 110°07′37″ W), collected between 1816 m and 2290 m above sea level, and Chiricahua, Arizona (31°54′55″ N; 109°15′95″ W), collected between 1963 m and 2567 m above sea level. A map of the collection sites has been provided (Fig. S1). The three populations were maintained as isolines, which are inbred descendants of a single female (David et al. 2005): 11 from Lewistown, 24 from Show Low, and seven from Chiricahua. Although some populations of *D. pseudoobscura* carry the sex ratio distorting X chromosome meiotic driver “*Sex Ratio*” or “*SR*”, we screened each isoline for *SR* and only used lines without the driver. At the start of each new generation, three virgin males and females of each isoline were placed in a standard *Drosophila* vial (25 × 75 mm) on a medium of rolled oats, brown sugar, dried yeast, agar, nipagin, and water (Shorrocks 1972). All flies were maintained at 23°C under a 14:10 h photoperiod, with lights on at 10:00 GMT. We transferred flies using a mouth pooter and did not anaesthetize them as this is known to disrupt copulation behavior (Barron 2000).

### Assays of propensity to remate

Female and male flies were collected and separated by sex within 18 h of eclosion so as to ensure virginity (Policansky 1979; Greenspan 1997). Both the initial mating assay, as well as the remating assay, was carried out at 23°C. Flies were sexed and transferred to fresh food in sex-specific vials of 20–30 flies per vial. Females were 4–5 days old, and males were 3–7 days old for the initial mating assay, to ensure sexual maturity (Beckenbach 1978; Snook and Markow 2001). Virgin males were placed in individual vials the day before the mating assay and left overnight to habituate. One female of the corresponding isoline as the male was transferred to each vial. Vials were observed continuously for two hours to record successful copulations, and the latency to copulation and copulation duration were recorded. Copulations that lasted less than a minute were deemed pseudocopulations and were removed from the assay. Following each mating, males were removed from the vials, and the females kept individually in an incubator at 23°C. After 4 days, these females were again paired with a male from their own isoline and observed for two hours as before. We established this as a good measure of propensity to remate from previous work carried out by Price et al. (2008). This is due to the fact that it correlates with the number of days to remating when a female is presented with a male every day for six consecutive days (range of mean day of remating: 2–5 days). Furthermore, it correlates with the remating frequency when 100 males and 100 females are placed in a large bottle simultaneously together 4 days after initially mating (Price et al. 2008). Importantly, this measure of remating propensity can be used as a proxy for polyandry, as it correlates well with population measures of polyandry, detected using genotyping of families (Price et al. 2014), and also correlates with polyandry in relatives in nature (Price et al. 2011). We were unable to produce enough male virgins as mating partners for each isoline, so a randomized mixture of sexually mature virgin and nonvirgin males was used for the remating assay. However, previous work has shown that female remating rate is predominantly under female genetic control (Price et al. 2008). Females that were observed to mate again were recorded, and in this way, the proportion of females that remated could be calculated for each isoline. A mean of 36 trials were successfully carried out for each isoline (range 17–65). One isoline from the Lewistown population had only one female that mated, so this isoline was removed from the experiment.

### Karyotype determination

**Figure 1:**
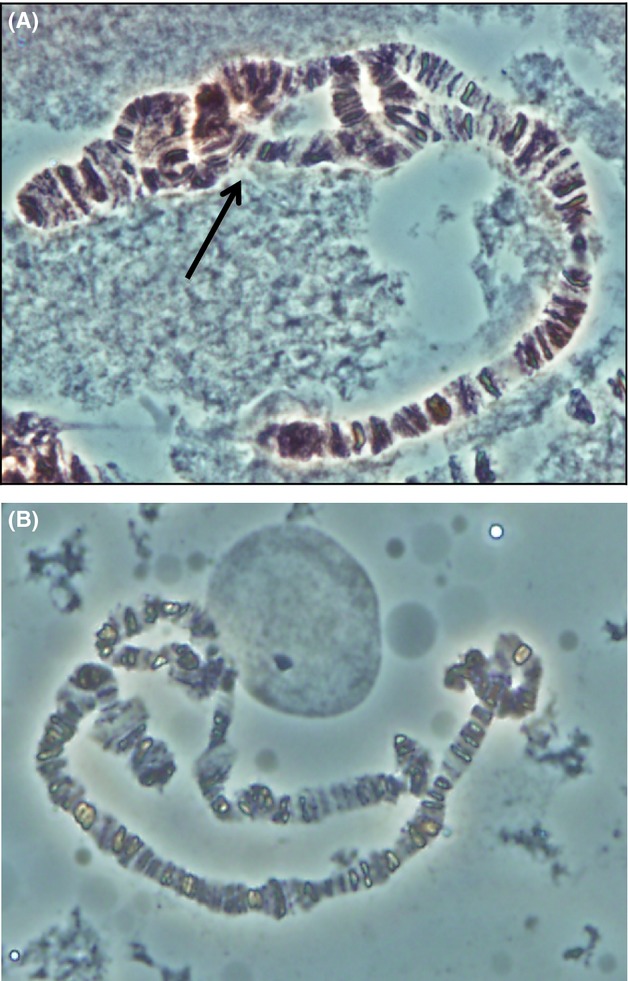
(A) Photograph taken of a chromosome squash of a larva from isoline E9 (Lewistown population), showing an inversion pattern of AR/TL (black arrow). (B) Photograph taken of a squash from isoline C4 (Show Low population), showing a homokaryotype, as no inversion loops are present. By crossing larvae from this isoline with flies homozygous for the standard karyotype, we now know that this isoline is homozygous for AR.

We determined the karyotype of each isoline by examining the giant chromosomes that occur in the salivary chromosomes of *Drosophila* larvae (Kastritsis and Crumpacker 1967). During meiosis I, chromosomes line up in homologous pairs, and the mismatch of DNA that occurs when an individual carries two different inversions causes distinctive loops to form. The salivary gland chromosomes of third instar larvae from each isoline were dissected in Ringer's solution and then fixed in acetic acid for five minutes. The glands were then stained with aceto-orcein for ten minutes. They were rinsed with acetic acid, and excess moisture blotted away before being covered with a cover slip and squashed to rupture the cell membranes and spread the chromosomes apart. The salivary gland squashes were observed at ×40 magnification with a compound microscope to make an initial assessment of whether larvae carried homozygous or heterozygous chromosomes (Fig. 1). Breakpoints of the inversions were identified by comparing photographs with a photographic key produced by Kastritsis and Crumpacker (1967) and used to identify which inversions were present in heterozygous individuals. Eight larvae from each isoline were assayed to increase the accuracy of identifying the chromosomal inversions present in the isoline. As homozygous individuals do not produce inversion loops during meiosis, hence, their inversion type could not be identified by squashing larvae from the isoline. We therefore crossed homozygous isolines to strains known to have the standard (i.e., no inversions) karyotype and karyotyped their offspring, allowing accurate determination of the inversion present on the third chromosome in the homozygous isolines.

### Statistical analyses

All data analysis was carried out using R version 2.15.2 (Ihaka and Gentleman 1996). For each isoline, all the trials were grouped together so as to provide a single measure of remating per isoline. Generalized linear models (GzLMs) and generalized linear mixed effect models (GzLMMs) were used to analyze the data except where stated. We constructed three GzLM models to examine, respectively, the following: (1) how does female remating vary with population when ignoring karyotype, (2) how does female remating vary among isolines when ignoring population, and (3) does the presence of heterokaryotypes differ between populations? GzLMMs were used when testing the relationship between female remating and presence of heterokaryotypes, as we wanted to remove the effect of the populations. Population was placed as a random effect as we wanted to examine whether, in general, female remating levels were correlated with the presence of heterokaryotypes, regardless of which population it originated from. MASS library was used in the case of GzLMs, and lme4 library was used in the case of GzLMMs. In both model types, we used the maximal model for each response variable and then used a stepwise removal of factors to produce a final minimal adequate model (Grafen and Hails 2002), using chi-square significance tests for the binomial models and an *F* test for the quasibinomial model. We used c-binded values to analyze the proportion of remating data appropriately as it allows R to use the logit transformation (log(*P*/1–*P*)). We used quasibinomial error structure to correct for overdispersion in the first GzLM that examined female remating with respect to population.

## Results

### Frequency of polyandry in each population

**Figure 2:**
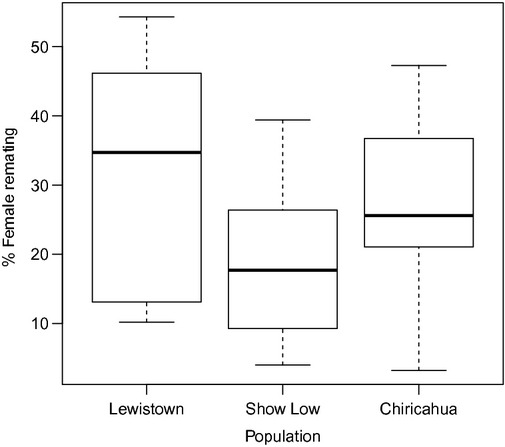
Box plot of the median proportion (and 25–75% interquartile) range of females that remated 4 days after their initial mating for each of the three populations. Lewistown is located furthest north, whereas Chiricahua is furthest south. Females from Lewistown remated the most, whereas females from Show Low were the least polyandrous.

Considerable variation in polyandry was observed within and between the different populations and isolines (Table 1). There was a significant difference in female remating rates among the different populations (GzLM with quasibinomial error structure, *F*_2,38_ = 3.291, *P *=* *0.048, Fig. 2), with populations possessing a mixture of homo- and heterokaryotypes (i.e., Lewistown and Chiricahua populations) exhibiting the highest levels of polyandry (Fig. 2). It should be noted that isolines that possessed heterokaryotypes were not necessarily absent of homokaryotypes, but a mixture of the two karyotypes in that particular isoline was the norm. Similarly, polyandry differed significantly among isolines (GzLM with binomial error structure, *χ*^2^_40_ = 157.19, *P *<* *0.001). This confirms that there is significant genetic variation in female remating propensity as previously reported for these populations (Price et al. 2014).

**Table 1:** Number of isolines examined in each population; minimum, maximum, and mean frequency of female remating per population and which inversions are present.

Population	No. of isolines	Remating rate (%)			Inversions present
		Minimum	Maximum	Mean	
Chiricahua	7	3.2	47.3	27.4	AR, CH, PP
Lewistown	10	10.2	54.3	32.2	AR, PP, TL
Show Low	24	4.0	39.4	18.2	AR

Inversions present: AR-Arrowhead; CH-Chiricahua; PP-Pikes Peak; and TL-Tree Line (Kastritsis and Crumpacker 1967).

### Polyandry and heterokaryotypes

A significant difference was observed between the presence of heterokaryotypes among the three populations (GzLM with binomial error structure, *χ*^2^_2_ = 20.135, *P *<* *0.001), with a clear distinction between the Lewistown and Chiricahua populations and that of Show Low, as all isolines of the latter population were homokaryotypic (Table 1). However, no relationship was found between frequency of female remating and the presence of heterokaryotypes within an isoline (GzLMM with binomial error structure and population as a random effect, *χ*^2^_1_ = 0.108, *P *=* *0.743). This analysis was repeated after removing all isolines from the Show Low population, as none of these possessed heterokaryotypes, but again no significant interaction was observed (GzLMM with binomial error structure and population as a random effect, *χ*^2^_1_ = 0.389, *P *=* *0.533).

### Chromosome inversion types and relationship to polyandry

**Figure 3:**
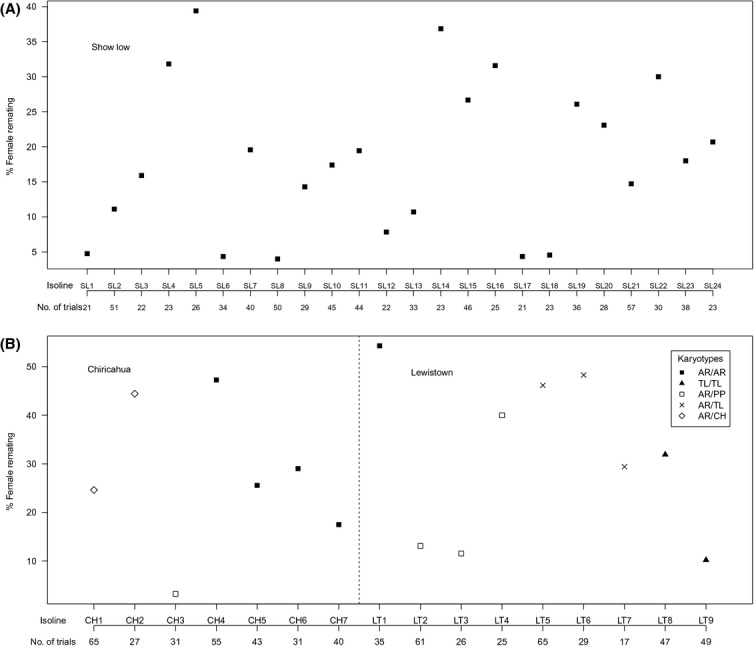
Scatter plot showing the proportion of females that remated from each isoline. (A) shows the 24 isolines from Show Low and (B) shows the proportion of females that remated in the seven isolines from Chiricahua and nine isolines from Lewistown along with their known karyotypes. Homo- and heterokaryotypes are denoted by the filled and open symbols, respectively.

We found that all 24 isolines from the Show Low population were homozygous for the Arrowhead (AR) inversion. In Chiricahua, four isolines were homozygous for Arrowhead, two were Arrowhead/Chiricahua heterokaryotypes, and one was an Arrowhead/Pikes Peak heterokaryotype. In Lewistown, one isoline was homozygous for Arrowhead, three were Arrowhead/Pikes Peak heterozygotes, three were Arrowhead/Treeline heterozygotes, and two were homozygous for Treeline. Figure 3A and B depict the karyotypes of the known isolines together with the female remating rates of each isoline. Examination suggests there is no clear association of polyandry with any karyotype or combination of karyotypes. Isolines homozygous for Arrowhead show almost the full range of rates of polyandry, from 4% remating to 54%. There is a possible trend for Treeline homozygotes or Treeline/Arrowhead heterozygotes to be more polyandrous, but the rates of polyandry are within the range shown by other karyotypes, and the sample size is too small for useful statistical analysis. Although the sample size of each karyotype is small, there is a trend that flies with the AR/PP (mean 17%) karyotype are less polyandrous than those possessing the AR/TL (mean 41%) or AR/CH (mean 35%) karyotypes. Unfortunately, we were unable to formally test any associations with specific karyotype due to the low sample size.

## Discussion

Females of most *Drosophila* species store a large amount of sperm after mating, which is used to fertilize the eggs as they are being laid. Remating usually results in sperm competition: the sperm from both males mix and compete within the female to fertilize the eggs. Ejaculates cause behavioral and physiological changes in the female, making her less willing to mate again for a period of time. These changes may include a decrease in attractiveness to males (Wolfner 1997), reduced receptivity to further mating (Fuyama 1995) as well as decreased lifespan (Chapman et al. 1995). Remating is common in females of numerous *Drosophila* species under both natural (e.g., Richmond and Powell 1970; Anderson 1974; Cobbs 1977) and laboratory conditions (e.g., Gromko and Pyle 1978; Singh and Singh 1999; Singh et al. 2002). Frequency of female remating, latency to remating, and duration of copulation during the first and second matings varies considerably among species (e.g., Smith 1956; Richmond and Ehrman 1974; Newport and Gromko 1984; Letsinger and Gromko 1985; McRobert et al. 1997; Singh and Singh 1999). These may be attributed to differences in the amount of sperm and seminal fluid that is transferred by males during copulation, as well as due to the varying female reproductive biology of the different species (Singh and Singh 2004).

Many hypotheses have been put forward to explain variation in frequency of polyandry in natural populations, but to date, little attention has been paid to the idea that female remating levels are associated with patterns of chromosome inversions. Inversion polymorphisms have previously been shown to be related to female mating activity in *D. pseudoobscura* (e.g., Spiess and Langer 1964; Parsons and Kaul 1966) and *D. subobscura* (Sperlich 1966), among other species, but it is not known if karyotypes are associated with variation in female remating frequency. In particular, the third chromosome polymorphism of *D. pseudoobscura* provides an opportunity to test the notion of genetic preservation of polyandry across geographic distributions, as chromosomes with different gene arrangements (i.e., inversion heterozygotes) may possess different gene complexes that provide different physiological and adaptive values (Dobzhansky and Epling 1948). Moreover, the separate gene arrangements may be coadapted in a specific way to yield higher adaptive levels of the inversion heterozygotes (Dobzhansky and Epling 1948). We used three natural populations of *D. pseudoobscura* collected from North America that differ in female remating frequency (Price et al. 2014) to determine whether polyandry can be explained by patterns of chromosome inversions across their geographic range. We found that mean female remating rates differed significantly between the three populations, being approximately 32% in Lewistown, 18% in Show Low, and 27% in Chiricahua. Lewistown, in Montana, is located around 1770 km north of Show Low and Chiricahua, both in Arizona. Previous work using flies from these populations has indicated that a latitudinal cline in polyandry exists across North America (Price et al. 2014). However, both the present study and Price et al. (2014) find consistent deviations by populations from this latitudinal cline. While there are some differences in female remating rate between the current and the Price et al. (2014) study using the same populations, qualitatively the pattern is similar. In both studies, polyandry is highest in the northern Lewistown population and lower in Show Low, but intermediate in Chiricahua, further to the south. This difference may be due to a lower number of female isolines (i.e., genotypes) examined in the current study than previously examined (Price et al. 2014).

Ideally, we would have had enough data for a rigorous statistical analysis of the association between each inversion and polyandry. Unfortunately, our limited sample of isolines and wide range of inversions means that we have too few samples for this to be feasible. Nevertheless, by simple inspection of the data, there was no clear relationship between the level of polyandry seen in an isoline and the karyotype of that isoline. Isolines homozygous for the commonest karyotype, Arrowhead, showed almost the full range of female remating rates, from 5% remating to 52%. There is a possible trend for Treeline to be associated with high levels of polyandry. However, the modest sample size of other karyotype combinations makes it difficult to evaluate this. Even if the Treeline inversion does carry alleles that cause high degree of polyandry, this cannot explain the difference in female remating rates between populations, because so much variation in frequency of polyandry occurs in non-Treeline isolines. Ideally, additional studies of a larger sample of isolines from additional populations that vary in polyandry would be useful to conclusively address this question.

As an alternative approach to test the relationship between polyandry and chromosome inversions, we investigated the broad scale interaction between polyandry and either the presence or absence of heterokaryotypes. Previous work has compared the relative fitness of polymorphic against monomorphic populations (Nickerson and Druger 1973), and heterozygote superiority (heterosis) has been reported on several occasions (e.g., Beardmore et al. 1960; Dobzhansky and Pavlovsky 1961; Pavlovsky and Dobzhansky 1966). Singh and Chatterjee (1988) also observed that inversion heterozygotes of *D. ananassae* males exhibited heterosis, as they had a higher mating propensity when compared to males with homozygote inversions. These results, as well as those of Singh and Chatterjee (1986), indicate that the male mating propensity parallels chromosome arrangement frequency in natural *D. ananassae* populations and that chromosomal polymorphism may have a partial behavioral basis. Similarly to *D. pseudoobscura* (Spiess et al. 1966), it appears that variation for mating propensity is considerably greater for male karyotypes than female karyotypes.

If heterozygotes are generally fitter, females may be more able to withstand any costs associated with polyandry. This could potentially explain why the Lewistown and Chiricahua populations demonstrated higher levels of polyandry than the Show Low population, as both showed a mixture of homo- and heterokaryotypes within the population. Despite this, we found no significant relationship between the presence of heterokaryotypes and female remating levels of the various isolines. However, the variation in homo- and heterokaryotypes among the populations is interesting and warrants further explanation as to what may be responsible for the maintenance of chromosomal polymorphism in a population. Superiority of inversion heterozygotes over the corresponding homozygotes is believed to be a crucial mechanism for preserving the gene arrangement polymorphism in *D. pseudoobscura* populations (Nickerson and Druger 1973). Nickerson and Druger (1973) evaluated hetero- and homokaryotypes relative to their respective fecundity, longevity as well as egg-to-adult viability and found heterozygote superiority for fecundity and longevity, but not for viability. The adaptive values of the karyotypes may also be dependent upon their relative frequencies (i.e., frequency-dependent selection) as well as the presence of certain other karyotypes within the same environment (Levene et al. 1954, 1958). Lewontin (1958) proposed that variable environments encourage the retention of polymorphism, and this may be lost if a population resides in a “constant” environment. Lewontin and Hubby (1966) suggested that for heterosis to be present to such a large extent, the adaptive superiority of heterozygotes must be explained for many different functions, only one of which may be polyandry.

An alternative explanation for polyandry in this species is that higher female remating rates reduce vulnerability to harmful selfish genetic elements (Price and Wedell 2008; Wedell 2013). *Sex Ratio* (*SR*) is a naturally occurring X chromosome meiotic driver that kills off the Y chromosome-bearing sperm of male carriers (Beckenbach 1981; Jaenike 2001) and causes significant ecological and evolutionary consequences by the production of female-biased populations. *SR* drive in *D. pseudoobscura* has a well-studied ecology, with *SR* being distributed in a latitudinal cline across North America, being absent in Canada and increasing in frequency further south, reaching its highest peak of 30% on the Mexican border (Wallace 1948; Dobzhansky 1958). Although the biological factors underlying geographical clines in *SR* frequency are not well-understood (Sturtevant and Dobzhansky 1936), polyandry can directly control the spread of *SR* in laboratory populations (Price et al. 2010). The large difference in levels of polyandry between the Lewistown and Show Low populations may explain the frequency of *SR* in these two locations – it is absent in Lewistown but high in Show Low (Price et al. 2014). However, we would expect a lower remating rate at Chiricahua as this is located so close to the Mexican border where *SR* has been recorded at 30% (Wallace 1948; Dobzhansky 1958).

This study has shown that separate populations of *D. pseudoobscura* exhibit different levels of polyandry. Populations also differed overall with respect to the presence of heterokaryotypes, and polyandry levels were greatest in the Lewistown and Chiricahua populations, which had a mixture of homo- and heterokaryotypes. In spite of this, there did not appear to be any direct relationship between the presence of heterokaryotypes and female remating levels of the various isolines. In conclusion, we find little evidence that the third chromosome polymorphism found in *D. pseudoobscura* is involved in the genetic control of polyandry. Although there is a previous report of female remating behavior being linked to a major inversion (Lawson et al. 2012), we find little evidence that the third chromosome polymorphism found in *D. pseudoobscura* is involved in the genetic control of polyandry.

## Supporting Information

**Figure S1.** Map of collection sites.
