# Supplementary figures and images for: Can patterns of chromosome inversions in Drosophila pseudoobscura predict polyandry across a geographical cline?
Source: Ecol Evol. 2014 Jul 10;4(15):3072–81. doi: 10.1002/ece3.1165 (PMC4161180; doi:10.1002/ece3.1165)

Supplementary Figure S1. Map of collection sites.


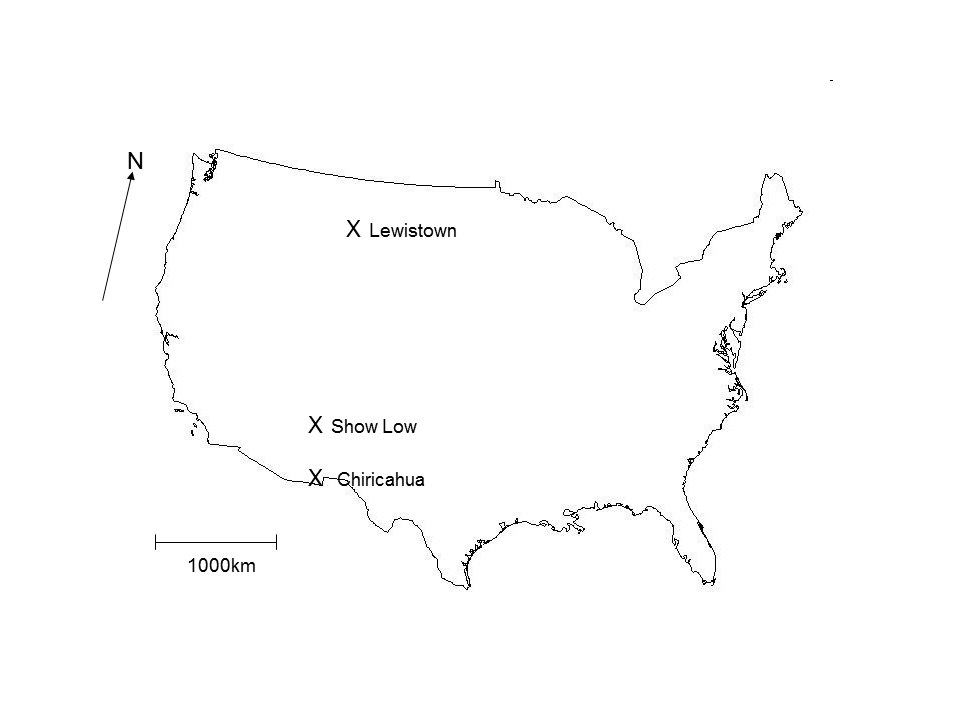

Supplement: Supplementary file 1 — Figure S1. Map of collection sites. [file ece30004-3072-sd1.docx]
